# Prediction of Radical Scavenging Activities of Anthocyanins Applying Adaptive Neuro-Fuzzy Inference System (ANFIS) with Quantum Chemical Descriptors

**DOI:** 10.3390/ijms150814715

**Published:** 2014-08-22

**Authors:** Changho Jhin, Keum Taek Hwang

**Affiliations:** Department of Food and Nutrition, Research Institute of Human Ecology, Seoul National University, Seoul 151-742, Korea; E-Mail: changho@jhin.kr

**Keywords:** QSAR, anthocyanin, semi-empirical, ANFIS, quantum chemical calculation

## Abstract

Radical scavenging activity of anthocyanins is well known, but only a few studies have been conducted by quantum chemical approach. The adaptive neuro-fuzzy inference system (ANFIS) is an effective technique for solving problems with uncertainty. The purpose of this study was to construct and evaluate quantitative structure-activity relationship (QSAR) models for predicting radical scavenging activities of anthocyanins with good prediction efficiency. ANFIS-applied QSAR models were developed by using quantum chemical descriptors of anthocyanins calculated by semi-empirical PM6 and PM7 methods. Electron affinity (*A*) and electronegativity (χ) of flavylium cation, and ionization potential (*I*) of quinoidal base were significantly correlated with radical scavenging activities of anthocyanins. These descriptors were used as independent variables for QSAR models. ANFIS models with two triangular-shaped input fuzzy functions for each independent variable were constructed and optimized by 100 learning epochs. The constructed models using descriptors calculated by both PM6 and PM7 had good prediction efficiency with Q-square of 0.82 and 0.86, respectively.

## 1. Introduction

**Figure 1:**
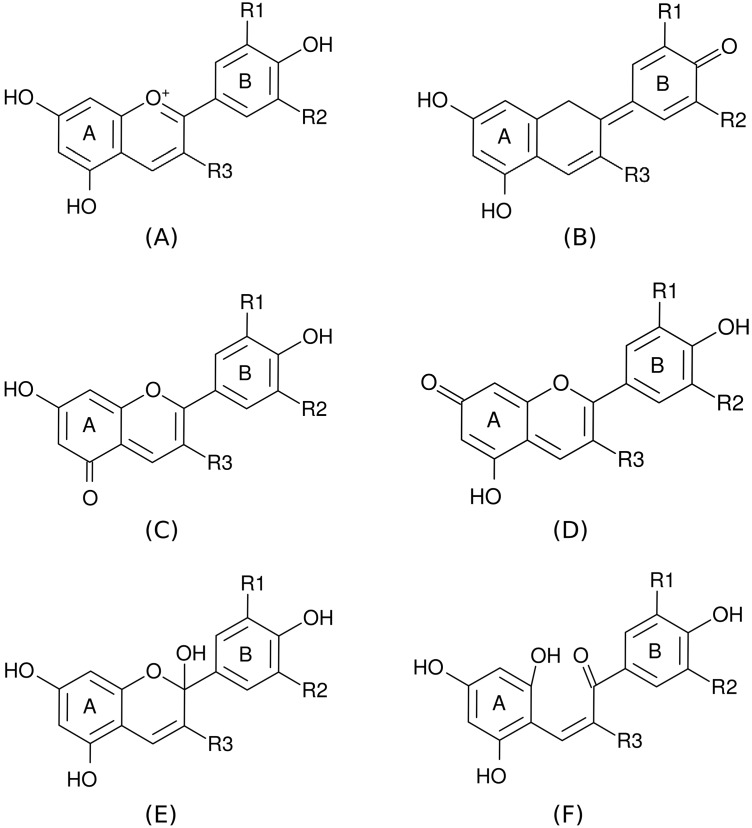
(**A**) Flavylium cation; (**B**–**D**) quinoidal bases; (**E**) carbinol pseudo base; (**F**) chalcone structure of anthocyanidin.

As constituents of flavonoid group, anthocyanins and their aglycones, anthocyanidins, are color pigments originated from plants. Anthocyanins basically have flavylium ion skeleton attached with different side groups including hydrogen atom, hydroxyl group, and methoxy group. Anthocyanins are classified by the position and type of the side groups (Figure 1 and Table 4).

Radical scavenging activity is one of the characteristics of anthocyanins and other flavonoids. These compounds can reduce reactive oxygen species (ROS), resulting in relieving oxidative stress [1,2]. The radical scavenging activities can be explained by two suggested mechanisms: One is hydrogen atom transfer (HAT) and the other is single electron transfer (SET) [3]. HAT-based radical scavenging activity is explained by hydrogen donation as following equation:

X + AH → XH + A
(1)

where X is a free radical, such as ROS, and AH is a molecule of antioxidant. Free radicals are scavenged by hydrogen atoms donated from an antioxidant. SET-based radical scavenging activity can be explained by the scheme below:

X + AH → X^−^ + AH ^+^ (2)

An electron from antioxidant transfers to a radical, and consequently the electron pairs up with unpaired electron of the radical. 2,2'-Azino-bis(3-ethylbenzothiazoline-6-sulphonic acid) (ABTS), a stable radical, is mainly reduced by SET mechanism [3,4], while 1,1-diphenyl-2-picryl hydrazyl (DPPH) radical is mainly reduced by HAT mechanism [5]. Until now, it has been considered that quantum chemical descriptors of ionization potential and bond dissociation energy are related with SET and HAT-based radical scavenging activities, respectively [6].

The relationship between molecular descriptors (e.g., number of hydroxyl group, presence of double bond on flavonoid C ring, and bond dissociation energy of hydrogen) of flavonoids and radical scavenging activities has been studied [6]. These prediction models can be applied to estimate and design novel radical scavenging bio-materials. However, the relationship between radical scavenging activities and molecular descriptors of anthocyanins has been poorly understood. Unlike other flavonoids, the structures of anthocyanins vary depending on pH [2,7]. At low pH (<2), flavylium cation (FC) is dominant with red and purple color. At higher pH, FC decreases while quinoidal base (QB), carbinol pseudobase (CP), and chalcone (Ch) forms of anthocyanins increase [7]. The change of the structure leads to the change of molecular descriptors of an anthocyanin molecule. For better understanding of the relationship between molecular descriptors of anthocyanin and radical scavenging activities, therefore, molecular descriptors of various anthocyanin forms should be considered.

To establish quantitative structure-activity relationship (QSAR) model, linear combination of variables have been generally used in previous studies [6,8]. The linear combination equation could be constructed easily through regression analysis. However, the interaction effect between variables is ignored, and nonlinear relationship between variables cannot be explained easily by linear combination. These kinds of defects may affect reliability of prediction model. Therefore, another prediction model is necessary for explaining the relationship between variables. Adaptive neuro-fuzzy inference system (ANFIS) is an artificial neural network (ANN)—applied fuzzy inference system (FIS). By using ANFIS, multi-variable related ambiguous relationship can be quantified by defuzzification process of FIS, and error is adjusted by backpropagation algorithm with hidden layer of ANN for reliable prediction [9]. Therefore, ANFIS is an effective technique for solving problems which cannot be easily solved by linear regression analysis. The aim of this study was to establish a QSAR model for predicting radical scavenging activities of anthocyanins by ANFIS using quantum chemical descriptors.

## 2. Results and Discussion

### 2.1. Correlation Analysis

The result of correlation analysis between DPPH radical scavenging activities and calculated Quantum chemical descriptors; which are ionization potential (*I*), electron affinity (*A*), chemical hardness (η), softness (*S*), electronegativity (χ), chemical potential (μ), electrophilicity (ω); of anthocyanin structures is presented in Table 1. Since 4 out of 21 tested anthocyanins had a glycosidic bond on 5-position, QB5 forms could not be generated. Thus, QB5 structures were excluded for further analysis. The χ and μ of FC, and *I* of QB7 calculated by both of PM6 and PM7 methods were significantly correlated with DPPH radical scavenging activities (*p* < 0.01). *A* calculated by PM6 and PM7 methods were also significant at *p* < 0.01 and *p* < 0.05, respectively. Some of the other quantum chemical descriptors of FC, QB7, and CP, calculated by PM6 and PM7 methods, were significant (*p* < 0.05). However, none of the quantum chemical descriptors of QB4' nor Ch was significantly correlated with radical scavenging activities. This result suggests that FC and QB7 forms are main contributors for radical scavenging characteristics of anthocyanins, rather than CP and Ch. Since the radical scavenging experiment by Kähkönen and Heinonen [10], of which data was adapted to this study, was not conducted on highly acidic condition nor on highly basic condition, QB and FC seemed to exist abundantly at experimental condition. The structural change affects quantum chemical descriptors and redox properties of molecules [8,11]. Borkowski, *et al.* [12] also reported that radical scavenging activities of anthocyanins were influenced by pH. In the case of QB, QB7 was suggested as a primary contributor for radical scavenging activities, because higher correlation was observed between quantum chemical descriptors of QB7 and radical scavenging activities compared with QB4'.

**Table 1:** Correlation coefficient between 1,1-diphenyl-2-picryl hydrazyl (DPPH) radical scavenging activities and quantum chemical descriptors of flavylium cation, quinoidal base, chalcone and carbinol pseudobase.

Descriptors	PM6					PM7				
	FC	QB4'	QB7	CP	Ch	FC	QB4'	QB7	CP	Ch
*I*	0.554 **	0.343	0.674 **	0.233	0.121	0.504 *	0.355	0.708 **	0.269	0.251
*A*	0.673 **	−0.166	−0.256	−0.473 *	−0.052	0.538 *	−0.206	−0.037	−0.289	−0.199
η	0.124	0.288	0.523 *	0.530 *	0.121	0.263	0.310	0.418	0.378	0.298
*S*	−0.153	−0.171	−0.503 *	−0.535 *	−0.138	−0.238	−0.132	−0.398	−0.411	−0.284
ω	0.328	−0.127	−0.305	−0.470 *	−0.054	0.202	−0.112	−0.085	−0.326	−0.212
χ	0.701 **	0.194	0.282	−0.344	0.009	0.567 **	0.087	0.551 **	−0.149	0.020
μ	−0.701 **	−0.194	−0.282	0.344	−0.009	−0.567 **	−0.087	−0.551 **	0.149	−0.020

FC: flavylium cation; QB7 and QB4': quinoidal bases (Figure 1B, D, respectively); CP: carbinol pseudobase; Ch: chalcone; *I*: ionization potential; *A*: electron affinity; η: chemical hardness; *S*: chemical softness; ω: electrophilicity; χ: electronegativity; and μ: chemical potential. *, ** significance (*p* < 0.05 or *p* < 0.01).

For further investigation, hydrogen atom bond dissociation energies (BDE) of FC and QB7 were calculated and compared. Some previous studies reported that negative relationship was observed between the lowest BDE (BDE_min_) and radical scavenging activities [6,8]. Physicochemically, hydrogen abstraction reaction is more favoured at the position with a lower BDE, because a hydroxyl group with a lower BDE needs less energy to dissociate hydrogen. In addition, the dissociated hydrogen pairs up with a free radical to scavenge it. For this reason, BDE_min_ represents the radical scavenging activity by HAT mechanism. Average BDE of FC and QB7 structures were lowest at the hydroxyl group on 4'-position (Table 2). This tendency was also observed in a previous study by density function theory method [13,14,15]. It has been reported that BDE_min_ of flavonoid was negatively correlated with radical scavenging activities by Amić and Lučić [8]. In this study, however, BDE_min_ of FC and QB7 calculated by PM6 and PM7 were not significantly correlated with DPPH radical scavenging activities (*p* > 0.05). This result was probably caused by conformational difference between anthocyanins, of which conformation changes depending on pH, and flavonoids. Thus, BDE was excluded for establishing QSAR model.

**Table 2:** Hydrogen atom dissociation energy of flavylium cations and quinoidal pseudo bases calculated by semi-empirical methods.

Compounds	Method		^(1)^ BDE of FL											BDE of QB7									
			3	5		7		3'		4'		5'		3		5		3'		4'		5'	
cyanidin	PM6		**80.98**	84.8		90.2		82.06		83.34		-		**67.08**		75.5		76.04		72.44		-	
	PM7		81.77	**81.77**		91.41		83.43		84.78		-		**68.98**		75.72		75.54		75.16		-	
delphinidin	PM6		78.15	84.24		90.53		83.16		**75.61**		78.55		**65.65**		74.8		79.17		68.74		74.47	
	PM7		82.55	85.75		92.2		85.58		**77.6**		82.08		**68.2**		75.42		78.74		71.29		75.13	
malvidin	PM6		77.33	82.05		87.19		-		**73.9**		-		**65.49**		74.83		-		68.04		-	
	PM7		81.19	85.22		90.98		-		**75.21**		-		**71.31**		78.69		-		73.36		-	
pelargonidin	PM6		**81.01**	84.79		90.39		-		89.53		-		**68.45**		75.5		-		74.43		-	
	PM7		**81.86**	85.71		91.51		-		90.6		-		**69.68**		75.89		-		76.87		-	
peonidin	PM6		80.86	84.58		90.45		-		**77.37**		-		**68.38**		76.99		-		69.2		-	
	PM7		**81.86**	85.71		91.51		-		90.6		-		**71.53**		78.3		-		73.51		-	
cyanidin-3-coumaroyl-sambubioside-5-galactoside	PM6		-	-		88.15		**80.05**		82.17		-		-		-		**74.12**		75.16		-	
	PM7		-	-		86.57		**77.29**		78.88		-		-		-		**76.74**		77.52		-	
cyanidin-3-sambubioside-5-galactoside	PM6		-	-		89.15		**80.07**		82.15		-		-		-		**74.12**		75.23		-	
	PM7		-	-		98.43		**88.66**		90.97		-		-		-		**76.74**		78.07		-	
cyanidin-3,5-diglucoside	PM6		-	-		91.23		**80.23**		83.08		-		-		-		**74.85**		75.92		-	
	PM7		-	85.22		-		**80.39**		82.39		-		-		-		**77.19**		78.28		-	
cyanidin-3-arabinoside	PM6		-	86.49		91.25		**80.53**		82.61		-		-		77.32		76.27		**73.99**		-	
	PM7		-	88.14		92.69		**82.63**		85.54		-		-		77.87		**77.09**		77.48		-	
cyanidin-3-galactoside	PM6		-	93.17		93.58		**81.03**		83.71		-		-		76.74		**74.76**		75.55		-	
	PM7		-	96.62		96.76		**84.86**		85.8		-		-		81.26		80.34		**79.3**		-	
cyanidin-3-glucoside	PM6		-	86.87		92.58		**81.78**		84.4		-		-		76.07		**74.91**		75.63		-	
	PM7		-	87.79		82.92		**82.92**		85.51		-		-		**77.07**		77.52		77.99		-	
cyanidin-3-rutinoside	PM6		-	88.51		94.22		**80.41**		83.25		-		-		76.6		**74.72**		76.12		-	
	PM7		-	90.49		94.22		**81.73**		84.65		-		-		80.23		**77.36**		78.9		-	
delphinidin-3-glucoside	PM6		-	86.92		91.58		82.36		**75.31**		77.35		-		76.9		78.53		**69.28**		74.42	
	PM7		-	89.94		92.99		84.51		**77.89**		80.94		-		80.64		80.53		**77.06**		80.23	
delphinidin-3-rutinoside	PM6		-	88.23		91.05		82.52		75.93		**75.74**		-		75.22		77.2		**70.95**		72.55	
	PM7		-	84.54		92.68		84.59		77.79		**76.34**		-		**72.62**		80.15		73.79		73.62	
malvidin-3,5-diglucoside	PM6		-	-		86.32		-		**73.67**		-		-		-		-		**71.07**		-	
	PM7		-	-		91.84		-		**76.05**		-		-		-		-		**72.45**		-	
malvidin-3-galactoside	PM6		-	87.49		89.43		-		**73.81**		-		-		74.52		-		**69.57**		-	
	PM7		-	88.43		91.56		-		**75.73**		-		-		78.65		-		**72.45**		-	
malvidin-3-glucoside	PM6		-	85.75		89.51		-		**73.89**		-		-		76.84		-		**68.59**		-	
	PM7		-	88.63		94.26		-		**78.17**		-		-		73.52		-		**71.67**		-	
pelargonidin-3-glucoside	PM6		-	87.13		91.13		-		**86.79**		-		-		**71.66**		-		75.48		-	
	PM7		-	**86.20**		93.35		-		89.08		-		-		**72.61**		-		78.55		-	
peonidin-3-galactoside	PM6		-	88.79		98.46		-		**76.39**		-		-		79.56		-		**68.31**		-	
	PM7		-	88.94		92.01				**78.78**		-		-		77.10		-		**72.78**		-	
peonidin-3-glucoside	PM6		-	88.80		90.97		-		**76.38**		-		-		74.66		-		**67.51**		-	
	PM7		-	88.57		91.51		-		**78.74**		-		-		78.95		-		**72.74**		-	
petunidin-3-glucoside	PM6		-	86.34		90.85		82.33		**74.17**		-		-		76.79		79.31		**68.40**		-	
	PM7		-	88.62		94.78		87.69		**78.51**		-		-		73.73		80.33		**71.51**		-	

^(1)^ BDE: hydrogen atom bond dissociation energy (kcal/mol); FL: flavylium cation; and QB7: quinoidal base (Figure 1D). The lowest BDE is presented in bold.

The possible mechanism for DPPH radical scavenging activities could be suggested by these results. Since BDE_min_ was not significantly correlated with radical scavenging activities, the hydrogen atom transfer directly from anthocyanin to DPPH radical might not be a main mechanism for radical scavenging. Osman [16] suggested that DPPH radical and polyphenol compounds form a complex as an intermediate. For anthocyanin, as polyphenol, intermediate forming reaction could be occurred. Furthermore, the hydrazyl moiety of DPPH radical has nucleophilic characteristic and FC has high *A* and χ. The intermediate forming step was suggested as reaction barrier, and this could explain why the DPPH radical scavenging activities were highly correlated with *A* and χ of FC. On the other hand, from the positive correlation with *I* of QB7 and radical scavenging activity, electron transfer from QB7 to other molecules possibly lowers radical scavenging activities. The possible mechanism is single electron transfer from QB7 to FC. It is easy for a QB7 molecule with low *I* to transfer an electron to other molecules. Single electron addition to FC neutralizes net charge of the molecule to zero, and this will lower the *A* and χ of the molecule. The required energy for intermediate forming reaction between anthocyanin and DPPH radical tends to be increased, consequently, total radical scavenging activity is decreased. Matsufuji, *et al.* [17] reported that the DPPH radical scavenging activities of anthocyanins were higher in acidic condition (pH 3) than in neutral condition (pH 7), and this tendency could also be explained by our suggested mechanism. Since electron transfer from QB7 to FC is suggested as decreasing total radical scavenging activities, low occurrence of QB7 and high occurrence of FC in acidic condition contribute to high DPPH radical scavenging activity.

### 2.2. Prediction of Radical Scavenging Activities of Anthocyanins

Through correlation analysis, four quantum chemical descriptors of *A*, χ and μ of FC, and *I* of QB7 were most highly correlated with radical scavenging activities. Among these descriptors, both χ and μ were drawn by the Equation (7), the correlation coefficient (*R*^2^) between these two variables was exactly 1. Hence, using both descriptors was unnecessary, and χ, one of the two descriptors, was used for establishing the QSAR model. Previous studies also reported that χ and μ were reliable descriptors correlated with biological activities [11,18]. In addition, it has been reported that *n*OH was significantly correlated with radical scavenging activities of flavonoids and anthocyanins [6,8]. Therefore, *n*OH was chosen as an independent variable. Consequently, *A* and χ of FC, *I* of QB7, and *n*OH were used as independent variables for establishing QSAR model. The calculated *A* and χ of FC, *I* of QB7, *n*OH, and experimental DPPH radical scavenging activities were used as training data set for ANFIS model training. Structure of established ANFIS models is illustrated inFigure 2.

**Figure 2:**
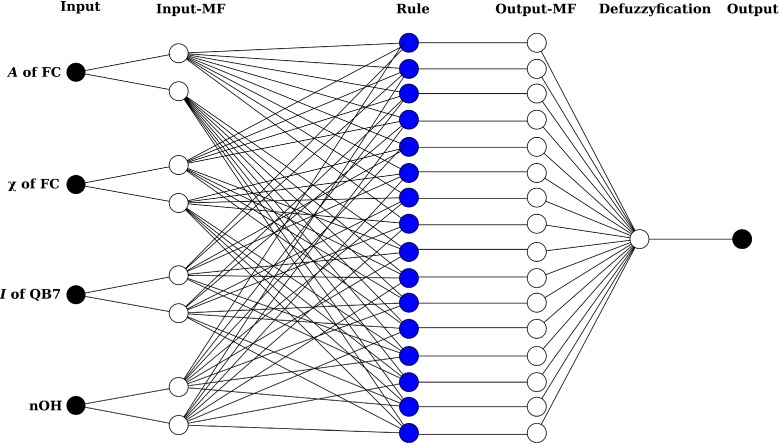
Developed adaptive neuro-fuzzy inference system (ANFIS) structure.

**Figure 3:**
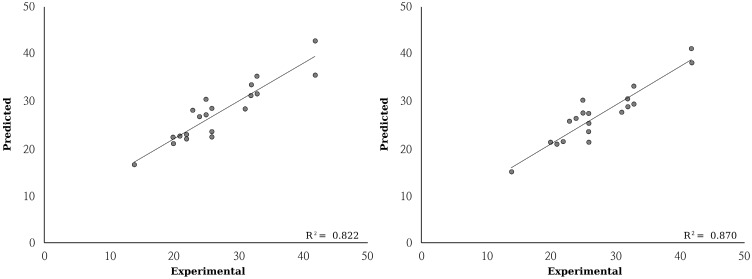
Relationship between predicted and experimental radical scavenging activities.

The experimental and predicted radical scavenging activities are presented in Table 3 and Figure 3. Since the number of naturally occurred and commercially available anthocyanins was limited, the test set with a relatively small number of compounds were only available to analyze. For small data set, cross validation with dividing data set into training set and test set might not be valid. Previous studies suggested a bootstrap validation method for small sample validation rather than cross validation [19,20]. Thus, bootstrap validation was performed in this study. The mean absolute error (MAE) and Q-square resulted by bootstrap validation were presented on Table 3. The ANFIS models with quantum chemical descriptors calculated by both of PM6 and PM7 semi-empirical methods had high prediction efficiency (*p* < 0.01). The error of the model continually decreased on every learning epoch (data not shown). After 100 learning epochs, the MAE of models with quantum chemical descriptors calculated by PM6 and PM7 were 2.44 and 2.06, respectively. Average DPPH radical scavenging activity of the tested compounds was 26.6. Thus, the prediction efficiency of established models was over 90%. Previous studies applied linear regression analysis for establishing QSAR models [6,11,18,19]. However, interaction between the variables was ignored in linear equations; therefore, a linear regression technique may not be suitable for multivariable analysis. Because four different independent variables were used in this study, linear regression analysis was not suitable for correcting interaction between variables. ANN has been applied on scientific studies because ANN can solve problems and predict problems practically as an adaptive intelligent system [9,20,21]. FIS can also solve non-linearly correlated variables by managing uncertainty of functions [22,23]. This explains the reason why ANFIS, an ANN applied problem-solving technique, was applied in this study. As a result, the established model showed high prediction efficiency and the ANFIS model fitted well with experimental data.

**Table 3:** Experimental and predicted DPPH radical scavenging activities.

Compounds	^(1)^ Experimental Radical Scavenging Activity	Predicted Radical Scavenging Activity	
		PM6	PM7
cyanidin	33	35.3	33.2
delphinidin	42	42.6	41.0
malvidin	24	26.6	26.1
pelargonidin	31	28.3	27.6
peonidin	33	31.4	29.3
cyanidin-3-coumaroylsambubiose-5-galactoside	26	22.4	25.2
cyanidin-3,5-diglucoside	21	22.6	20.9
cyanidin-3-arabinoside	26	28.5	27.3
cyanidin-3-sambubiose-5-galactoside	22	22.1	21.5
cyanidin-3-galactoside	25	30.3	30.2
cyanidin-3-glucoside	32	31.1	28.8
cyanidin-3-rutinoside	25	27.0	27.5
delphinidin-3-glucoside	42	35.4	38.1
delphinidin-3-rutinoside	32	33.4	30.4
malvidin-3,5-diglucoside	14	16.6	14.9
malvidin-3-galactoside	22	22.9	21.3
malvidin-3-glucoside	26	22.6	21.2
pelargonidin-3-glucoside	20	21.0	21.2
peonidin-3-galactoside	20	22.2	21.1
peonidin-3-glucoside	26	23.6	23.5
petunidin-3-glucoside	23	28.0	25.7
^(2)^ Mean absolute error		2.43 ± 0.35	2.06 ± 0.32
^(2)^ Q-square		0.82 ± 0.08	0.86 ± 0.08

^(1)^ Experimental radical scavenging activity data was adapted from a previous study by Kähkönen and Heinonen (2003), percentage of scavenged DPPH radical; ^(2)^ Mean absolute error and Q-squre are resulted from bootstrap validation, presented as mean ± standard deviation.

## 3. Experimental Section

### 3.1. DPPH Radical Scavenging Activity

DPPH radical scavenging activity data of 5 anthocyanidins and 16 anthocyanins were adapted from a previous study, which are listed in Table 4 [10].

**Table 4:** Evaluated anthocyanidins and anthocyanins.

Family	Compounds	^(1)^ *n*OH	R1	R2	^(2)^ R3	^(2)^ R4
anthocyanidin	cyanidin	5	OH	H	OH	OH
	delphinidin	6	OH	OH	OH	OH
	malvidin	4	OCH_3_	OCH_3_	OH	OH
	pelargonidin	4	H	H	OH	OH
	peonidin	4	OCH_3_	H	OH	OH
anthocyanin	cyanidin-3-coumaroyl-sambubioside-5-galactoside	3	OH	H	coumaroyl -sam	gal
	cyanidin-3-sambubioside-5-galactoside	3	OH	H	sam	gal
	cyanidin-3-arabinoside	4	OH	H	ara	OH
	cyanidin-3-galactoside	4	OH	H	gal	OH
	cyanidin-3-glucoside	4	OH	H	glc	OH
	cyanidin-3-rutinoside	4	OH	H	rut	OH
	cyanidin-3,5-diglucoside	3	OH	H	glc	glc
	delphinidin-3-glucoside	5	OH	OH	glc	OH
	delphinidin-3-rutinoside	5	OH	OH	rut	OH
	malvidin-3-galactoside	3	OCH_3_	OCH_3_	gal	OH
	malvidin-3-glucoside	3	OCH_3_	OCH_3_	glc	OH
	malvidin-3,5-diglucoside	2	OCH_3_	OCH_3_	glc	glc
	pelargonidin-3-glucoside	3	H	H	glc	OH
	peonidin-3-galactoside	3	OCH_3_	H	gal	OH
	peonidin-3-glucoside	3	OCH_3_	H	glc	OH
	petunidin-3-glucoside	4	OH	OCH_3_	glc	OH

^(1)^ *n*-OH: number of hydroxyl groups on flavonoid core; ^(2)^ sam: sambubioside; gal: galactoside; ara: arabinoside; glc: glucoside; and rut: rutinoside.

### 3.2. Quantum Chemical Descriptors

#### 3.2.1. Molecular Structure Preparation

Figure 1 presents various structures of anthocyanidins and anthocyanins. (**A**)–(**F**) are FC, quinoidal bases (QB4', QB5, and QB7), CP, and Ch, respectively. The structures of 21 anthocyanidins and anthocyanins were prepared using Gabedit 2.4.3 [24].

#### 3.2.2. Calculation of Quantum Chemical Descriptors

Geometrical optimization of molecular structures was performed by semi-empirical methods of PM6 [25] and PM7 [26] using MOPAC2012 [27]. Quantum chemical descriptors of *I*, *A*, η, *S*, χ, μ, ω, and BDE were calculated to establish QSAR models.

*I* was calculated as the following equation:

*I* = *E*(*N* − 1) − *E*(*N*) (3)

where *E*(*N* − 1) is energy of an anthocyanin radical generated after electron abstraction and *E*(*N*) is energy of an anthocyanin molecule. *A* was calculated as the following equation:

*A* = *E*(*N*) − *E*(*N* + 1) (4)

where *E*(*N* + 1) is energy of an anthocyanin radical generated after addition of an electron.

η was calculated from the following equation:

η = (*I* − *A*)/2 (5)

*S* was calculated from the following equation:

*S* = 1/2η (6)

χ and μ were calculated from the following equation:

χ = −μ = (*I* + *A*)/2 (7)

ω was calculated from the following equation:

ω = μ^2^/2η (8)

Hydrogen atom dissociation energy (BDE) was calculated from the following equation:

BDE = *E*(A–O·) + *E*(H) − *E*(A–OH) (9)

where *E*(A–O·) is energy of a hydrogen abstracted anthocyanin phenoxyl radical, *E*(H) is the energy of a hydrogen atom, and *E*(A–OH) is energy of anthocyanin molecule.

### 3.3. QSAR Model Development

#### 3.3.1. Correlation Analysis

Pearson’s correlation analysis was conducted to examine the relationship between the DPPH radical scavenging activities and the quantum chemical descriptors using GNU R 3.0.1 [28]. The quantum chemical descriptors which were highly correlated with radical scavenging activities were chosen as independent variables for QSAR models.

#### 3.3.2. ANFIS

ANFIS models were made using a fuzzy logic toolbox of Matlab 8.2 (Mathworks, Natick, MA, USA). Previous studies reported that the number of phenolic OH groups of flavonoid core (*n*-OH) was correlated with radical scavenging activities of flavonoids [8] and anthocyanins [19]. Therefore, *n*-OH, as well as quantum chemical descriptors, was used for establish QSAR model development.

For development of ANFIS models, *A*, and χ of FC, *I* of QB7, and *n*OH were used as independent variables. DPPH radical scavenging activities of anthocyanins were used as a dependent variable. Two triangular-shaped membership functions for each independent variable, 16 if-then rules and 16 linear type output membership functions were developed (Figure 2). The ANFIS models were optimized by backpropagation method with 100 learning epochs.

To validate the constructed ANFIS models, bootstrap validation procedure was repeated 1000 times. Mean absolute error (MAE) was calculated and compared by bootstrapping. MAE was calculated from the following equation:

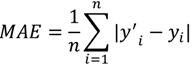
(10)
where *y′_i_* is predicted DPPH radical scavenging activity and *y_i_* is experimental one.

Also, Q-square between predicted radical scavenging activities and experimental ones was calculated and validated by bootstrap validation.

## 4. Conclusions

Semi-empirical quantum chemical calculations of anthocyanins were done by PM6 and PM7 methods using MOPAC2012. PM7 is the most recently distributed semi-empirical calculation method. This is the first study regarding anthocyanins using PM7 method. This study suggests that PM7, as well as PM6, is a useful method for calculating quantum chemical descriptors for QSAR analysis. In addition, in this study, structural change of an anthocyanin was considered on semi-empirical quantum chemistry calculation. This study revealed that quantum chemical descriptors of flavylium cation and quinoidal base affect radical scavenging activities of anthocyanins. It suggests that the molecular conformation should be modified depending on surrounding condition before quantum chemical calculation. Established QSAR models by ANFIS showed good prediction efficiency with a statistical significance. Therefore, applying the ANFIS technique could improve the accuracy of QSAR models. However, the QSAR analysis of this study was limited to radical scavenging activities of anthocyanins. Further study is needed to verify the correlation between quantum chemical descriptors and biological activities of anthocyanins.
